# Effect of culture conditions on growth, lipid content, and fatty acid composition of *Aurantiochytrium mangrovei* strain BL10

**DOI:** 10.1186/2191-0855-2-42

**Published:** 2012-08-10

**Authors:** Kai-Chuang Chaung, Chun-Yao Chu, Yu-Ming Su, Yi-Min Chen

**Affiliations:** 1 Institute of Biotechnology, National Cheng Kung University, Tainan, Taiwan; 2 Vedan Enterprise Corporation, Tainan, Taiwan; 3 Center of Bioscience and Biotechnology, National Cheng Kung University, Tainan, Taiwan

**Keywords:** Lipid, Polyunsaturated fatty acid (PUFA), Docosahexaenoic acid (DHA), *Aurantiochytrium mangrovei*, Thraustochytrid

## Abstract

This study explored the influence of various culture conditions on the biomass, lipid content, production of docosahexaenoic acid (DHA), and fatty acid composition of *Aurantiochytrium mangrovei* strain BL10. The variables examined in this study include the species and concentration of salt, the concentrations of the two substrates glucose and yeast extract, the level of dissolved oxygen, the cerulenin treatment, and the stages of BL10 growth. Our results demonstrate that BL10 culture produces maximum biomass when salinity levels are between 0.2 and 3.0%. Decreasing salinity to 0.1% resulted in a considerable decrease in the biomass, lipid content, DHA production, and DHA to palmitic acid (PA) (DHA/PA) ratio, signifying deterioration in the quality of the oil produced. The addition of 0.9% sodium sulfate to replenish salinity from 0.1% to 1.0% successfully recovered biomass, lipid content and DHA production levels; however, this also led to a decrease in DHA/PA ratio. An increase in oxygen and cerulenin levels resulted in a concomitant decrease in the DHA to docosapentaenoic acid (DPA) (DHA/DPA) ratio in BL10 oil. Furthermore, the DHA/DPA and DHA/PA ratios varied considerably before and after the termination of cell division, which occurred around the 24 hour mark. These results could serve as a foundation for elucidating the biochemistry underlying the accumulation of lipids, and a definition of the extrinsic (environmental or nutritional) and intrinsic (cell growth stage) factors that influence lipid quality and the production of DHA by BL10.

## Introduction

Recently, docosahexaenoic acid (DHA) has received considerable attention for its benefits to human health (Bruce and Julie 2002; Riediger et al. 2009). DHA is an important fatty acid in brain and retina (Muskiet et al. 2004). DHA plays a critical role in the neural development of fetuses and infants, as well as in the maintenance of brain function during adulthood (Muskiet et al. 2004; Singh 2005). This fatty acid is crucial to the prevention of dementia, depression, and many diseases affecting the cardiovascular system (Bruce and Julie 2002). Humans obtain DHA mainly from food, and fish oil has traditionally been the primary source (Muskiet et al. 2004). However, over exploitation of marine resources and accumulation of persistent organic pollutants in aquatic environments has increased the demand for alternative sources of DHA (Sijtsma and Swaaf 2004). Because the DHA found in fish can be traced back to microalgae in lower trophic levels of the marine ecosystem (Sijtsma and Swaaf 2004), it is reasonable to consider marine microalgae as a potential source of DHA. However, making this proposal financially feasible would require industrial scale production of DHA through high density cultivation of microphytes. Current applications now focus on two categories of microalgae: *Crypthecodinium cohnii*, the major DHA source for infant formula fortification, and thraustochytrids, which provides lower-cost oils with multiple polyunsaturated fatty acids (PUFAs), are used as human dietary supplements and additives in animal feed ( Spolaore et al. 2006). *Schizochytrium* is the major species of thraustochytrid used in the production of DHA (Kamlangdee and Fan 2003; Fan et al. 2001; Yaguchi et al. 1997). However, other thraustochytrid species such as *Thraustochytrium* (Burja et al. 2006; Bajpai et al. 1991) and *Ulkenia* (Kiy et al. 2005) are currently being investigated in order to evaluate their suitability for these applications.

BL10 is an *Aurantiochytrium mangrovei* (formally called *Schizochytrium mangrovei* (Yokoyama and Honda 2007)) strain isolated from an estuary close to Taipei City, Taiwan. It is an oleaginous microalgal strain. Under the optimal conditions established in our previous research, this organism produced dry biomass of 59 gL^-1^. Dry biomass was comprised of 73% fatty acids, nearly 40% of which was DHA (29% of dry biomass) (Yang et al. 2010). The fatty acid content and DHA yield of most strains of thraustochytrid are not particularly susceptible to the influence of salinity; however, reducing the salinity from 3.0% to 1.0% induced a dramatic increase in the oil production of BL10 (from 58% to 73% of dry biomass). In addition, the productivity of BL10 is not affected by high glucose levels (140 gL^-1^), which can lead to the retardation of growth in other thraustochytrid strains (Yaguchi et al. 1997; Burja et al. 2006). These results revealed unique physiological characteristics of BL10 cells, including adaptability to environmental change, the regulation of fatty acid synthesis, and the accumulation of lipids.

The aim of this study was to elucidate the unique characteristics of BL10 from a cell physiology perspective, to identify the bio-function of various fatty acids in BL10, and to determine how to optimize the production of DHA in this microalgal strain. Thus, we performed a comprehensive investigation to determine how intrinsic and extrinsic factors influence the biomass, fatty acid composition, total fatty acid (TFA) content, DHA production, and oil quality of BL10. Specifically, the extrinsic factors included salinity, salt species, concentration of substrates (glucose (Glc) and yeast extract (YE)), oxygen levels, as well as the effect of adding the antibiotic cerulenin (a fungal antibiotics specifically binds and inhibits β–ketpacyl-ACP synthetase in fatty acid synthase complex (Omura 1976)) to the medium, which has demonstrated useful to selectively block the synthesize of saturated fatty acid in *Schizochytrium* strain ATCC20888 (Hauvermale et al. 2006). Intrinsic factors included the growth stage of BL10 culture.

Docosapentaenoic acid (DPA, C22:5n-6) and palmitic acid (PA, C16:0) are other major fatty acids present in BL10 cells; however, these substances are considered undesirable. As a structural analog and antagonist of DHA, the uptake of DPA may result in an unfavorable loss of DHA in the brain (Lim et al. 2005), while an excessive uptake of PA could increase the risk of developing cardiovascular disease (Zock et al. 1994). Thus, this study adopted DHA/DPA and DHA/PA ratios as the criteria of oil quality.

## Materials and methods

### BL10 cultivation

BL10 (BCRC980009) was maintained through monthly sub-cultivation on plates containing H3 medium (composed of 1 gL^-1^ peptone, 2 gL^-1^ YE, 4 gL^-1^ Glc; dissolved in full-strength seawater with additional 0.8% agar) at a 20.0 ± 0.1°C incubator. A single colony (0.3-0.5 cm in diameter) cultivated on a plate for less than one week was picked up by a loop, and transferred to a 50-mL test tube (with a screw-cap) containing 5-mL sterile H3 medium for the preparation of a seed culture. The cap was loosened (screwed tight and subsequently turned counterclockwise 360 degrees) and fixed with a piece of tape in order to prevent detachment. The tube was then fixed on a rack, tilted at a 50-degree angle, and shaken at 150 rpm in a 27.0 ± 0.1°C incubator, without aeration, for 48 hours. Aliquots (50 μL) of the seed culture were then transferred to 50-mL test tubes with 5-mL of media prepared specifically for the proceeding studies.

Media for the assay of salinity and salt species was produced by dissolving YE/Glc (9/90 gL^-1^) in various dilutions of seawater with salinity values of 3.0, 1.5, 1.0, 0.5, 0.2, and 0.1%; or in 1.0% artificial seawater (made by adding either 0.9% sodium chloride or sodium sulfate to seawater diluted to 0.1%). The effects of YE/Glc on BL10 were examined at concentrations of 4.5/45, 9/90, 9/120, and 9/150 g of dissolved substrate per L^-1^ of 1.0% artificial seawater with sodium sulfate.

To examine the effects of growth stage, oxygen level, and the addition of cerulenin, we used a basal medium containing YE/Glc (4.5/45gL^-1^) dissolved in 1.0% artificial seawater (composed of 0.1% diluted seawater and 0.9% sodium sulfate). Eight microliters of either pure ethanol or ethanol solution containing various concentrations of cerulenin were added to the basal medium in order to obtain final cerulenin concentrations of 0, 1, 5, and 25 μM.

The effects of salinity, salt species, substrate enrichment, oxygen level, and the addition of cerulenin were examined in 18 replicate tubes for each treatment. Three tubes from each treatment were randomly selected every 24 hours, harvested by centrifuging (3 min at 4,000 × g in a 15-mL centrifugation tube) or filtering through a glass-fiber filter (pore size 1.6-μm, 2.5 cm in diameter). The biomass yield was then rinsed with 2-mL distilled water to remove salts and the residual substrate, and then lyophilized and weighed to determine the growth stage of the culture. When the dry biomass of the culture plateaued, the cells were collected and analyzed for fatty acid content and composition.

To examine the influence of oxygen level, we employed three different treatments: open (O), open-closed (O-C), and closed (C). The cap of the O-group was kept loosened (turned 360 degrees counterclockwise immediately after being screwed tight) throughout the entire period of cultivation; the cap of the O-C group was unscrewed for the initial 12 hrs and screwed tightly thereafter; and the cap of the C-group was screwed tightly for the entire period of cultivation.

The effects of growth stage were examined in 30 replicate tubes. To determine cell density and fatty acid composition, as well as glucose and oxygen levels in the medium, 3 tubes were randomly selected at 6, 12, 18, 24, 36, 48, 72, 96, and 120 hours following inoculation. Immediately after removing the cap of each tube, the oxygen level was measured using an EcoScan DO6 oxygen meter (Eutech, Singapore), in accordance with the manufacturer’s instructions. Ten micro-liters of the culture medium was divided and transferred onto a hemocytometer (Marienfeld, Germany) to measure cell density. The remainder of the culture medium was harvested and rinsed to obtain clean cell pellets, which were subsequently lyophilized to determine biomass and fatty acid composition and content. Using the dinitrosalicylic acid (DNS) reagent, we also tested the supernatant obtained during harvesting in order to calculate residual glucose levels in the medium (Miller 1959).

### Biomass determination and fatty acid analysis

The lyophilized cell pellets were weighed to determine the biomass (cell dry weight per unit volume, gL^-1^) of BL10 culture. The fatty acid composition and content in the dry biomass of BL10 were determined according to the protocols established in our previous investigation (Yang et al. 2010).

### Statistical analysis

Data related to DHA/DPA and DHA/PA ratios were analyzed using one-way ANOVA (Microsoft Excel 2010), with p >0.05 considered statistically significant.

## Results

### Salinity and salt species

**Figure 1:**
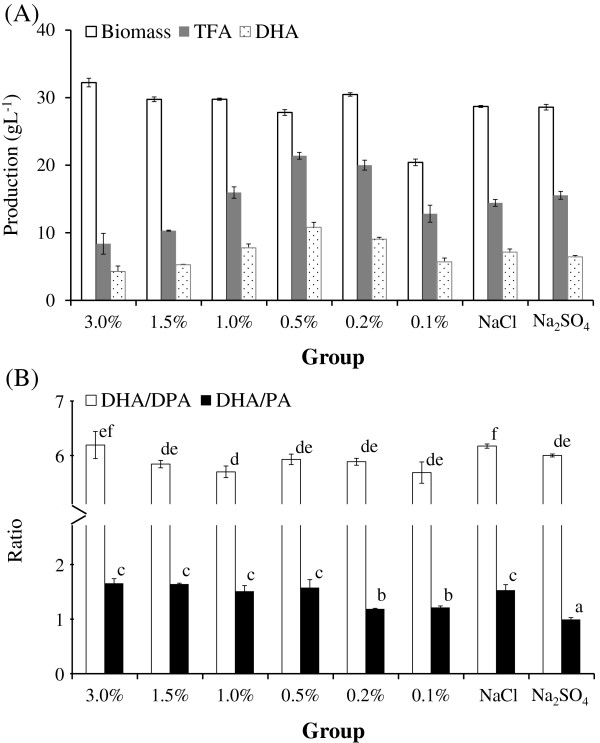
The effect of salinity and salt species on (A) biomass, TFA content, DHA production of BL10 culture; and on (B) quality of oil produced by BL10.

As illustrated in Figure 1 (A), BL10 grew well and maintained a stable biomass across a wide range of salinity levels (from 0.2% to 3.0%). Conversely, decreasing salinity from 0.2 to 0.1% resulted in a considerable decrease in biomass. However, when we supplemented the salt content of 0.1% diluted seawater solution with either 0.9% sodium chloride or sodium sulfate, biomass yield recovered to levels comparable with those cultivated at salinity levels exceeding 0.2%.

The biomass remained stable over a wide range of salinity levels; however, the fatty acid content was strongly influenced by changes in salinity. As illustrated in Figure 1 (A), TFA content and DHA production of BL10 was positively correlated with a decrease in salinity, reaching a plateau at 0.5%, and gradually dropping with salinity levels below 0.5%. Replenishing salt content in the medium through the addition of either 0.9% sodium chloride or sodium sulfate into 0.1% diluted seawater recovered the TFA content and DHA production to the same level as which cultivated in 1.0% diluted seawater.

Salinity and salt species had a greater influence on the DHA/PA ratio than on the DHA/DPA ratio (Figure 1 (B)). A slight (but not significant) decrease in DHA/PA ratio was observed when salinity was decreased from 3.0% to 0.5%; however, the decrease became significant when salinity dropped from 0.5 to 0.2%. The addition of 0.9% sodium chloride (the main salt species in natural seawater) successfully recovered the DHA/PA ratio; however, the addition of 0.9% sodium sulfate resulted in a further decrease in the DHA/PA ratio.

The decrease in DHA/PA at salinity levels of 0.1% and at salinity levels of 0.1% with additional 0.9% sodium sulfate can be attributed to a decrease in the percentage of DHA and a concomitant increase in the percentage of PA in TFA, as shown in Table 1.

**Table 1:** **The Influence of salinity and salt species on the fatty acid composition of BL10**^***a***^

**Salinity (%)**	**Fatty acid composition (% of TFA)**						
	**C14:0**	**C15:0**	**C16:0 (PA)**	**C17:0**	**C18:0**	**C22:5n-6 (DPA)**	**C22:6n-3 (DHA)**
**3.0**	4.3 ± 0.4	2.9 ± 0.2	31.5 ± 1.3	0.6 ± 0.0	0.7 ± 0.1	8.2 ± 0.2	48.6 ± 3.0
**1.5**	3.2 ± 1.5	3.1 ± 0.6	27.4 ± 4.6	0.5 ± 0.1	0.6 ± 0.1	7.8 ± 1.5	45.3 ± 8.0
**1.0**	4.5 ± 0.4	3.6 ± 0.2	32.2 ± 1.1	0.6 ± 0.0	0.8 ± 0.1	8.5 ± 0.3	48.7 ± 1.7
**0.5**	3.8 ± 0.3	2.9 ± 0.2	31.9 ± 1.1	0.5 ± 0.0	0.8 ± 0.0	8.4 ± 0.2	50.7 ± 1.6
**0.2**	2.1 ± 0.1	3.4 ± 0.6	37.5 ± 1.1	0.9 ± 0.1	0.9 ± 0.0	7.9 ± 0.2	45.1 ± 0.3
**0.1**	2.5 ± 0.1	5.3 ± 0.5	36.0 ± 1.7	1.5 ± 0.2	0.8 ± 0.0	7.9 ± 0.3	44.5 ± 0.4
**0.1% +0.9% Na**_**2**_**SO**_**4**_	4.1 ± 0.3	3.2 ± 0.2	42.3 ± 1.5	0.8 ± 0.0	1.1 ± 0.1	6.8 ± 0.3	40.8 ± 1.2
**0.1% +0.9% NaCl**	2.1 ± 0.2	3.7 ± 0.3	32.2 ± 1.1	0.5 ± 0.1	0.6 ± 0.0	8.0 ± 0.2	49.4 ± 1.5

^*a*^Results are represented as mean ± SD.

Commercial production of thraustochytrid may require the replacement of salts in natural seawater through the addition of sodium sulfate to maintain optimal growth conditions, prevent the formation of cell clumps, and reduce the corrosion of stainless steel fermenters (Raghukumar 2008). Thus, we employed this formula as a part of our basal medium in the following experiments, despite the negative influence it may have on quality of the oil produced by BL10.

### Glc and YE concentrations

**Figure 2:**
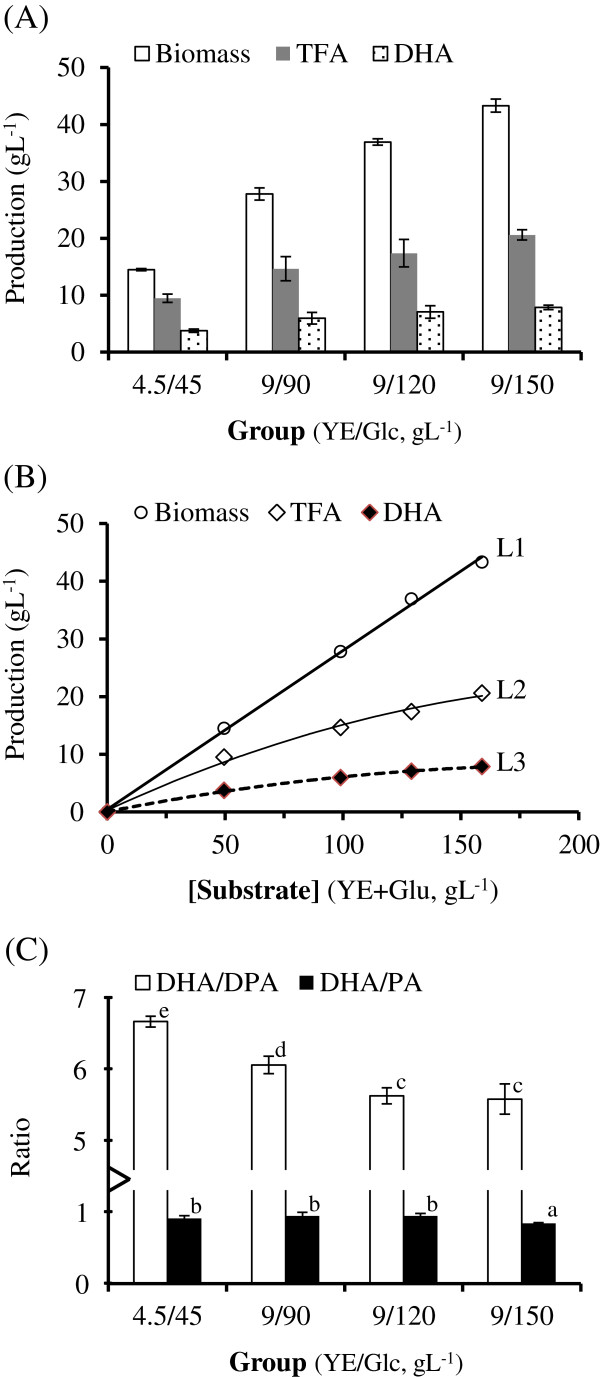
(A) The effects of substrate concentrations on the biomass, TFA content and DHA production of BL10 culture; (B) Correlations between total substrate concentration and biomass, TFA content, and DHA production of BL10. The formulas used to determine the curve of best fit are presented in the Results section; (C) The effect of substrate concentrations on quality of oil produced by BL10.

The biomass, TFA content, and DHA production of BL10 culture concomitantly increased with an increase in the substrate concentration of the medium, as illustrated in Figure 2 (A).

To determine whether the biomass, TFA content, and DHA production increased linearly with an elevation in substrate concentration (total concentration of YE and Glc), we employed regression analysis to identify correlations between the substrate concentration and biomass, TFA content, and DHA production respectively (Figure 2 (B)). The formulas used to determine the curve of best fit between substrate concentration (X) and biomass (Y_1_), TFA content (Y_2_) and DHA production (Y_3_) are $L1:Y_{1}=0.2753\;X+0.4660\;\left(R^{2}=0.9983\right)$, $L2:Y_{2}=0.000{4X}^{2}+0.1882X+0.3188\;\left(R^{2}=0.9943\right)$, and $L3:Y_{3}=-0.000{2X}^{2}+0.0807\text{X}+0.0702\;\left(R^{2}=0.9983\right)$. The results show that only biomass, yet TFA content and DHA production increased linearly in elevated substrate concentration; the higher the substrate concentration, the lower the conversion rate from substrate to fatty acids.

An elevated substrate concentration resulted in not only a decrease in the fatty acid conversion rate, but also detraction from the quality of the oil produced. As shown in Figure 2(C) and Table 2, the DHA/PA and DHA/DPA ratios and DHA percentage decreased concomitantly with an increase in substrate concentration. Thus, in the following experiments, we used YE/Glc = 4.5/45 gL^-1^ (the treatment with the lowest substrate concentration) as our basal ingredients in the medium.

**Table 2:** **The effect of substrate concentrations on the fatty acid composition of BL10**^***a***^

**YE/Glc (gL**^**-1**^**/gL**^**-1**^**)**	**Fatty acid composition**						
	**(% of TFA)**						
	**C14:0**	**C15:0**	**C16:0 (PA)**	**C17:0**	**C18:0**	**C22:5n-6 (DPA)**	**C22:6n-3 (DHA)**
**4.5/45**	5.4 ± 0.3	3.5 ± 0.1	43.5 ± 1.1	0.5 ± 0.0	0.7 ± 0.0	6.0 ± 0.2	39.6 ± 0.8
**9/90**	4.2 ± 0.3	3.1 ± 0.0	42.8 ± 11.7	0.8 ± 0.0	1.0 ± 0.0	6.7 ± 0.3	40.5 ± 1.7
**9/120**	3.9 ± 0.3	3.0 ± 0.3	42.8 ± 0.5	0.8 ± 0.1	1.1 ± 0.0	7.2 ± 0.3	40.5 ± 0.9
**9/150**	4.1 ± 0.2	2.9 ± 0.2	45.4 ± 0.3	0.8 ± 0.0	1.1 ± 0.1	6.9 ± 0.2	38.2 ± 0.2

^*a*^Results are represented as mean ± SD.

### Growth stages

**Figure 3:**
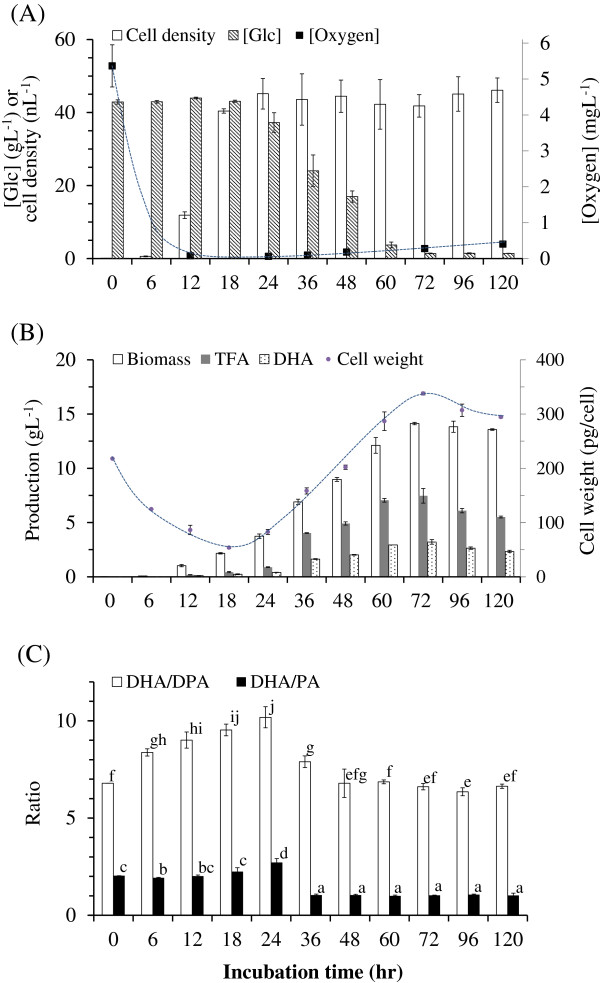
Time course of (A) cell density, glucose concentration, and oxygen level of BL10 culture; (B) cell size (weight per cell), biomass, TFA content, and DHA production of BL10 culture; and (C) quality of oil produced by BL10.

The number of cells in BL10 culture increased rapidly between the initiation of incubation and the 18 hour mark, which was inversely correlated with a significant decrease in oxygen levels from the initial 6 mgL^-1^ to near zero at 18 hrs (Figure 3 (A)). Rapid cell division resulted in a considerable drop in weight per cell. Individual cell weight had decreased from the initial 217 pg/cell to 53 pg/cell at the 18 hour mark. This was followed by a considerable increase in glucose consumption between 18 and 72 hrs (Figure 3 (B)). This consumption of glucose is clearly unrelated to cell division because cell density had nearly plateaued at 18 hrs (Figure 3 (A)). Rather, the elevated glucose consumption can be attributed to the synthesis of fatty acids, which resulted in a concomitant augmentation of lipid content, as well as weight per cell: from 53 pg/cell at 18 hrs to 338 pg/cell at 72 hrs (Figure 3 (B)).

The content of nonpolar lipids as a percentage of total lipids increased from 12% at 18 hrs to 94% at 72 hrs (preliminary data). These results revealed that the fatty acid produced following the termination of cell division was mainly used in the synthesis of nonpolar lipids.

The DHA/DPA and DHA/PA ratios varied considerably before and after the termination of cell division, which occurred around the 24 hour mark. Our observations suggest that oil quality improved during cell division; and subsequently plummeted following the termination of cell division (initiation of fatty acid accumulation). In addition to changes in the relative quantity of the three major fatty acids (DHA, PA, and DPA), we observed significant change in the percentage of the two linear fatty acids with odd-numbered carbon: C17:0, and particularly C15:0, near the termination of cell division (Table 3). C15:0 and C17:0 increased from 5.0% and 0.7% (as a percentage of TFA) at 12 hrs, to 27.3% and 3.0% at 24 hrs respectively. However, these concentrations rapidly dropped to basal levels at 48 hrs.

**Table 3:** **Time course of fatty acid composition during cultivation of BL10**^***a***^

**Time (hr)**	**Fatty acid composition**						
	**(% of TFA)**						
	**C14:0**	**C15:0**	**C16:0 (PA)**	**C17:0**	**C18:0**	**C22:5n-6 (DPA)**	**C22:6n-3 (DHA)**
**0**	2.6 ± 0.4	5.0 ± 0.5	26.7 ± 3.4	2.3 ± 0.0	0.7 ± 0.3	8.0 ± 0.3	54.5 ± 3.7
**6**	2.0 ± 0.6	4.0 ± 1.0	27.8 ± 5.1	1.2 ± 0.3	0.7 ± 0.3	5.6 ± 0.6	47.1 ± 1.2
**12**	2.2 ± 0.5	5.0 ± 0.5	28.1 ± 4.7	0.7 ± 0.0	0.5 ± 0.3	6.3 ± 0.7	56.1 ± 4.5
**18**	1.7 ± 0.1	11.1 ± 0.2	24.2 ± 1.3	1.7 ± 0.1	0.4 ± 0.1	5.7 ± 0.0	54.2 ± 1.8
**24**	1.7 ± 0.1	27.3 ± 0.8	16.7 ± 1.2	3.0 ± 0.2	0.2 ± 0.1	4.5 ± 0.5	45.2 ± 0.4
**36**	5.1 ± 0.2	7.3 ± 0.6	39.1 ± 0.9	0.9 ± 0.0	0.6 ± 0.0	5.2 ± 0.4	40.6 ± 1.2
**48**	4.8 ± 0.3	4.8 ± 0.1	39.7 ± 1.1	0.8 ± 0.0	0.8 ± 0.1	6.1 ± 0.7	41.2 ± 0.5
**60**	4.5 ± 0.3	3.2 ± 0.2	41.8 ± 0.7	0.6 ± 0.0	0.9 ± 0.0	6.1 ± 0.2	41.7 ± 1.0
**72**	4.1 ± 0.1	2.9 ± 0.1	41.2 ± 0.3	0.6 ± 0.0	1.0 ± 0.0	6.4 ± 0.2	42.5 ± 0.2
**96**	3.6 ± 0.1	2.6 ± 0.1	40.7 ± 0.4	0.6 ± 0.0	1.1 ± 0.0	6.8 ± 0.1	43.3 ± 0.7
**120**	3.7 ± 0.2	2.6 ± 0.1	41.8 ± 2.2	0.6 ± 0.1	1.0 ± 01	6.4 ± 0.3	42.5 ± 2.6

^*a*^Results are represented as mean ± SD.

### Oxygen level

**Figure 4:**
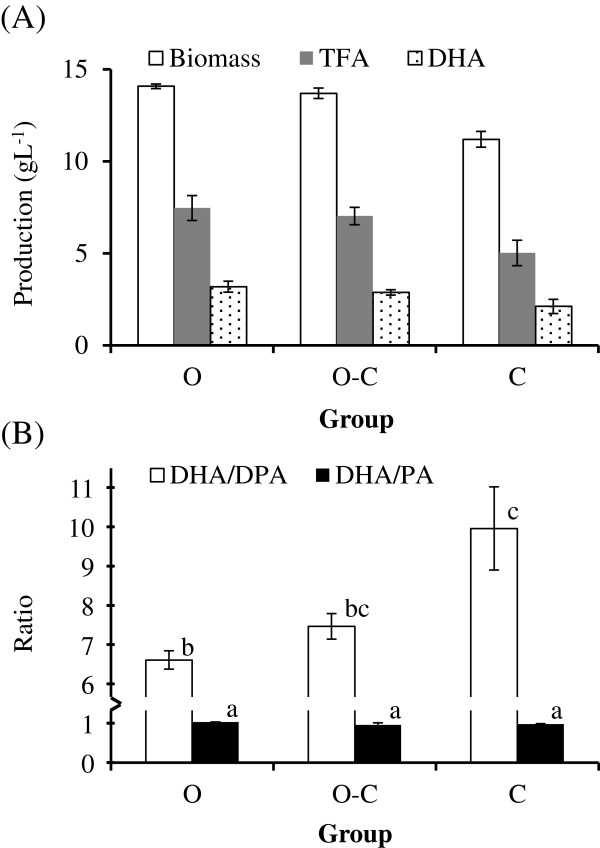
Effect of oxygen levels on (A) biomass, TFA content, and DHA production of BL10 culture; and on (B) quality of oil produced by BL10. The definitions of O, O-C, and C groups are presented in the Materials and Methods section.

Limiting the oxygen supply (beginning at 12 hrs) had little influence on biomass, TFA content, DHA production, or the oil quality of BL10. However, limiting oxygen at the initiation of incubation resulted in lower biomass, TFA content, and DHA production, as well as an elevated DHA/DPA ratio (Figure 4), attributed to a decrease in the percentage of DPA in TFA (Table 4) compared to other treatments. 

**Table 4:** **Effect of oxygen levels on fatty acid composition of BL10**^***a***^

**Groups**^*b*^	**Fatty acid composition**						
	**(% of TFA)**						
	**C14:0**	**C15:0**	**C16:0 (PA)**	**C17:0**	**C18:0**	**C22:5n-6 (DPA)**	**C22:6n-3 (DHA)**
**O**	4.2 ± 0.0	3.1 ± 0.1	42.1 ± 1.6	0.6 ± 0.0	1.0 ± 0.1	6.4 ± 0.3	41.2 ± 1.8
**C**	5.7 ± 1.9	2.2 ± 0.1	43.5 ± 0.6	0.4 ± 0.0	0.7 ± 0.0	4.2 ± 0.6	41.9 ± 1.9
**O-C**	5.0 ± 0.2	3.4 ± 0.1	41.6 ± 0.1	0.7 ± 0.0	1.1 ± 0.0	5.5 ± 0.2	40.9 ± 0.6

^*a*^Results are represented as mean ± SD.

^*b*^The definitions of O, O-C, and C groups are presented in the Materials and Methods section.

### Cerulenin treatment

**Figure 5:**
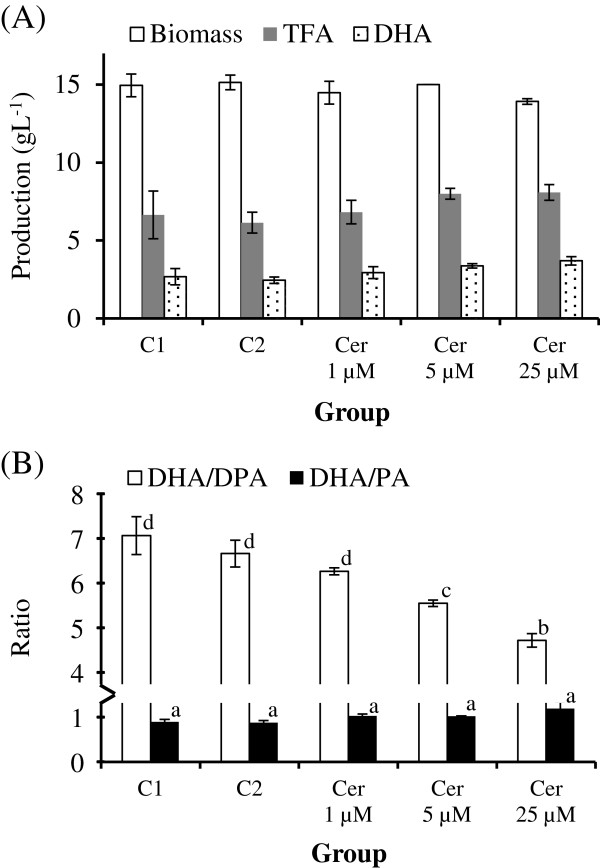
**Effect of cerulenin treatment on (A) biomass, TFA content, and DHA production of BL10 culture; and on (B) quality of oil produced by BL10.** C1 is negative control 1 (no cerulenin or ethanol); C2 is negative control 2 (with ethanol), Cer 1 μM, Cer 5 μM, and Cer 25 μM are three experimental groups treated with 1, 5, and 25 μM of cerulenin, respectively.

Increasing the cerulenin concentration in the medium resulted in the decreases in the percentages of the two even-numbered saturated fatty acids: C14:0 and particularly C16:0, but increased in the percentages of the two unsaturated fatty acids: DHA, and particularly DPA. Increasing the cerulenin concentration in the medium also resulted in a concomitant increase in DHA production, fatty acid content of BL10, and DHA/PA ratio (not significantly different), but a decrease in DHA/DPA ratio (Figure 5), which can be attributed to a more considerable increase in percentage of DPA than DHA under cerulenin treatment (Table 5). 

**Table 5:** **Effect of cerulenin treatment on the fatty acid composition of BL10**^***a***^

**Groups**^*b*^	**Fatty acid composition**						
	**(% of TFA)**						
	**C14:0**	**C15:0**	**C16:0 (PA)**	**C17:0**	**C18:0**	**C22:5n-6 (DPA)**	**C22:6n-3 (DHA)**
**C1**	4.0 ± 0.0	2.3 ± 0.2	45.2 ± 1.2	0.4 ± 0.0	1.0 ± 0.0	5.7 ± 0.1	40.6 ± 1.5
**C2**	4.2 ± 0.2	2.3 ± 0.1	45.4 ± 1.3	0.4 ± 0.0	0.9 ± 0.0	6.0 ± 0.4	40.0 ± 1.0
**Cer 1 μM**	4.0 ± 0.1	2.2 ± 0.0	41.7 ± 0.9	0.4 ± 0.0	0.9 ± 0.0	6.9 ± 0.2	43.1 ± 0.8
**Cer 5 μM**	4.1 ± 0.0	2.5 ± 0.0	41.1 ± 0.1	0.5 ± 0.0	0.9 ± 0.0	7.6 ± 0.1	42.2 ± 0.1
**Cer 25 μM**	3.7 ± 0.4	2.6 ± 0.2	35.5 ± 0.2	0.6 ± 0.2	0.8 ± 0.1	9.7 ± 0.4	46.1 ± 0.8

^*a*^Results are represented as mean ± SD.

^*b*^C1 is negative control 1 (no cerulenin or ethanol carrier), C2 is negative control 2 (with ethanol), Cer 1 μM, Cer 5 μM, and Cer 25 μM are three experimental groups treated with 1, 5, and 25 μM of cerulenin, respectively.

## Discussion

This investigation provided useful information for improving the production of DHA and the quality of oil from BL10 through the optimization of culture conditions. The first method is the adoption of 0.5% diluted seawater for the cultivation of BL10. In our study, cultures cultivated by 0.5% diluted seawater yielded the greatest production of high-quality oil in BL10 cells. This approach would require fermenters with anti-corrosion coating, because chloride levels in 0.5% diluted seawater are highly corrosive to stainless steel. In addition, the decrease in dry biomass and increase in cellular debris we observed with this treatment (compared with BL10 cultivated in 1.0% and 0.2%) suggests that the extremely high quantities of oil accumulating in cells could result in cell disruption. As a result, strong agitation that could shear cells must be avoided when lipid content is high. For cases in which anti-corrosion fermenters are unavailable, sodium sulfate could be used as an alternative to sea salts in order to reduce chloride ions in the medium. Unfortunately, the addition of sodium sulfate reduces the quality of the oil produced as well. To salvage the quality of oil, the fed-batch method (instead of the batch cultivation method) could be employed.

This investigation also provided useful information related to using BL10 microalgae as bioreactors to produce specific lipids, such as DHA-containing phospholipids, which have unique biotechnological value in the enrichment of live prey to improve the growth of fish larvae (Gisbert et al. 2005), and as a food supplement to reduce the risk of developing dementia (Schaefer et al. 2006). Our preliminary data shows that, after 18 hours of growth, 88% of the total lipids harvested from cells were phospholipids, in which 62% of the fatty acid was composed of DHA. These results could serve as a protocol for the production of DHA-containing phospholipids from BL10 culture. A similar concept which used a glucose-deficient medium to stimulate the conversion of DHA-rich triacylglycerol to DHA-containing phospholipids in a thraustochytrid-like microorganism (strain B12) has been mentioned in previous study (Okuyama et al. 2007).

Prior research has investigated the environmental conditions that influence the composition of fatty acids and the accumulation of lipids in strains of *Aurantiochytrium,* and concluded the following:

1. Temperature: In a study on *Aurantiochytrium* sp. strain mh0186, Taoka et al (2009) discovered that an elevation in temperature from 10°C to 35°C results in a concomitant decrease in both DHA/DPA and DHA/PA ratios. Furthermore, in an investigation of *Aurantiochytrium* sp. strain OUC-88, Zhu et al (2007) reported an increase in the production of the two linear and odd-numbered fatty acids C15:0 and C17:0 at higher temperatures (37°C).

2. Salinity and salt species: Yaguchi *et al* (1997) determined that the optimal salinity concentration for the cultivation of *Aurantiochytrium limacinum* strain SR21 to achieve maximum biomass ranges between 50% and 200% salinity of sea water. These results are similar to those found for *Aurantiochytrium* strain OUC-88 (Zhu et al. 2007). In addition, the lipid content in OUC-88 increased slightly from 41.34 to 48.97%, following a decrease in salt concentration from 3.6 to 0.9%. Palmitic acid accounted for 43.38% of the TFA when this microalga was cultivated at 3.5%, and dropped to 32.62% at a salinity level approaching 0%. However, a different pattern in the production of fatty acids was observed in *Aurantiochytrium mangrovei* strain Sk-02, which has low TFA content in a weak saline environment (between 0 and 1.0%), and higher, stable TFA content in strong saline environment (between 1.0 and 6.8%) (Unagul et al. 2006).

3. Substrate concentration: Glucose levels as high as 100 gL^-1^ resulted in the retardation of growth in *Aurantiochytrium* strains MP2 (Wong et al. 2008) and SR21 (Yaguchi et al. 1997).

4. Growth stage: In a study of *Aurantiochytrium* sp. strain T66, Jakobsen et al (2008) revealed that the DHA/DPA ratio is slightly higher in the lipid-accumulation phase than in the cell division phase. Chi et al (2009) later reported that the cell size of *A. limacinum* strain SR21 is stable during cell division, but continually increases during the lipid accumulation stage.

5. Oxygen level: Jakobsen et al (2008) suggested that lipid accumulation can be initiated by limiting the availability of O_2_, which also results in an increase in the DHA/DPA ratio. Chi et al (2009) further reported that aeration has a negative influence on the accumulation of fatty acids in *A. limacinum* strain SR21.

6. Response to cerulenin: Hauvermale et al. (2006) investigated the influence of cerulenin on the synthesis of fatty acids in *Schizochytrium* sp. strain ATCC20888 (our preliminary study suggested that it could be re-classified into *Aurantiochytrium* due to its close phylogenetic relationship with other known *Aurantiochytrium* strains). This study revealed a significant reduction in the synthesis of saturated fatty acids when cerulenin levels exceeded 1 μM. Furthermore, increasing cerulenin levels to greater than 25 μM resulted in a near total blockage in the synthesis pathways of both saturated and non-saturated fatty acids.

This study discovered considerable differences between BL10 and other *Aurantiochytrium* strains with regard to the synthesis of fatty acids and the accumulation of lipids, including the following:

1. Response to salinity and salt species: The optimal salinity for the production of biomass and DHA by BL10 is 0.5%, which is considerably lower than that of other strains of *Aurantiochytrium* (Yaguchi et al. 1997; Zhu et al. 2007). In addition, the effect of salinity on fatty acid production differs considerably as well (Zhu et al. 2007; Unagul et al. 2006), since the known strains of *Aurantiochytrium* are not particularly susceptible to the influence of salinity; however, reducing the salinity could induce a dramatic increase in the oil production of BL10. Given that BL10 is capable of maximum DHA production in a weaker saline environment (which is less corrosive to stainless steel fermenters), BL10 is actually a more promising candidate for the commercial production of DHA.

2. Substrate concentration: increasing glucose levels to as high as 150 gL^-1^ did not influence the growth rate of BL10. Thus, BL10 has a greater tolerance for high glucose levels in the culture medium than other species of *Aurantiochytrium* (Yaguchi et al. 1997; Wong et al.^ 2008^).

3. Growth stages: BL10 has a considerably higher DHA/DPA ratio during the cell division phase than during the lipid accumulation phase. This trend is the opposite of that observed in *Aurantiochytrium* strain T66 (Jakobsen et al. 2008). A continuous decrease in the size of BL10 during cell division stage also differs from that of strain SR21, which maintains stable cell size throughout the stage (Chi et al. 2009).

4. Tolerance to cerulenin: BL10 is clearly more tolerant to cerulenin treatment than ATCC20888 (Hauvermale et al. 2006). Treatment with 25 μM cerulenin arrested the synthesis of fatty acids in ATCC20888; however, this treatment had far less influence on the fatty acid composition and DHA production of BL10.

Our results revealed a number of other interesting characteristics related to the composition of fatty acids and the production of lipids by BL10 in response to environmental changes, including the following:

1. The influence of sodium sulfate on DHA/PA ratio.

2. Dramatic up-regulation in the synthesis of C15:0 and C17:0 when cell division approached termination. The up-regulation in the synthesis of C15:0 and C17:0 was first reported for *Aurantiochytrium* cultivated under conditions of high temperature and low salinity (Zhu et al. 2007), revealing the possible anti-stress role of the two fatty acids. An increase in the two fatty acids in BL10 toward the termination of cell division is much more significant than under conditions of low salinity; therefore, we speculate that the two fatty acids may play a more important role in shifting the condition of cell physiology from cell proliferation in the cell division stage to energy storage in the lipid accumulation stage, and since synthesis of C15:0 and C17:0 needs a precursor: propionate, a metabolic intermediate from degradation of various amino acids, such as valine and isoleucine as well as α-ketobutyric acid (the metabolic products of methionine and threonine) (Smith and Macfarlane 1997; Vlaeminck et al. 2006), as a result, it has been suggested that there is a sudden increase in the turnover of intrinsic proteins as the cell division stage approaches termination, followed by an increase in concentration of propionate and the final up-regulation in the synthesis of C15:0 and C17:0.

## Competing interests

The author declares that they have no competing interests.
